# Comparative evaluation of cell-free tumor DNA in blood and disseminated tumor cells in bone marrow of patients with primary breast cancer

**DOI:** 10.1186/bcr2404

**Published:** 2009-09-21

**Authors:** Heidi Schwarzenbach, Klaus Pantel, Birthe Kemper, Cord Beeger, Friedrich Otterbach, Rainer Kimmig, Sabine Kasimir-Bauer

**Affiliations:** 1 Institute of Tumor Biology, University Medical Center Hamburg-Eppendorf, Martinistrasse 52, Hamburg, 20246, Germany; 2 Department of Gynecology and Obstetrics, University of Duisburg-Essen, Sabinastrasse 20, Essen, 45136, Germany; 3 Institute of Pathology, University of Duisburg-Essen, Sabinastrasse 20, Essen, 45136, Germany

## Abstract

**Introduction:**

The origin and clinical relevance of circulating cell-free tumor DNA in the blood of cancer patients is still unclear. Here we investigated whether the detection of this DNA is related to bone marrow (BM) micrometastasis and tumor recurrence in breast cancer patients.

**Methods:**

BM aspirates of 81 primary breast cancer patients were analyzed for the presence of disseminated tumor cells (DTC) by immunocytochemistry using the pan-cytokeratin antibody A45-B/B3. PCR-based fluorescence microsatellite analysis was performed for detection of loss of heterozygosity (LOH) at 6 polymorphic markers using cell-free serum DNA. The data were correlated with established risk factors, and patients were followed-up over 6-10 years.

**Results:**

LOH was detected in 33.5% of blood samples. The occurrence of LOH at the entire microsatellite marker set correlated with histopathology (*P *= 0.05) and grading (*P *= 0.006) of the primary tumor. The genomic region characterized by marker D9S171 was only affected by LOH in patients with increased tumor stages (pT2-4, *P *< 0.05) and older age (≥ 55 years, *P *= 0.05). Kaplan-Meier analysis showed that LOH at D3S1255 (*P *= 0.009) and D9S171 (*P *= 0.001) were significantly associated with tumor relapse. In BM, DTC were detected in 39.5% of the patients, and this finding correlated with distant metastases (*P *< 0.05). Patients with DTC-positive BM had higher DNA yields in their blood than patients with DTC-negative BM (*P *< 0.05). However, no significant correlations were found between the presence of DTC in BM and the detection of marker-specific LOH on blood DNA.

**Conclusions:**

The detection of LOH on cell-free tumor DNA in blood is unrelated to BM micrometastasis and provides independent information on breast cancer progression.

## Introduction

Early hematogenous dissemination of tumor cells is a common phenomenon in breast cancer, which escapes detection by common staging procedures and limits the improvement of breast cancer mortality rates. In this regard, the spread of disseminated tumor cells (DTC) into the bone marrow (BM) is recorded in up to 40% of breast cancer patients at primary diagnosis, and their presence is being considered as an independent prognostic factor for reduced survival, as demonstrated by a pooled analysis of more than 4700 breast cancer patients [1]. Furthermore, DTC have been shown to persist in BM after conventional adjuvant chemotherapy (even after high-dose chemotherapy), and this persistence was associated with a worse prognosis [1-7]. Nevertheless, the detection of minimal residual disease (MRD) needs to be improved by additional factors because many BM-negative patients still relapse [4].

One of these factors might be cell-free DNA which is discharged during tumorigenesis from apoptotic and necrotic cells of the primary tumor into peripheral blood of patients with diverse tumor entities, including breast cancer [8-10]. Also, an active release of DNA by intact cells has been discussed [11]. Our recent study on cell-free DNA in blood from prostate cancer patients suggested that this DNA may also be originate from micrometastatic lesions [12]. This finding provided the rationale for the current study, which evaluates whether the detection of tumor-specific DNA in the blood of breast cancer patients is related to the presence of BM micrometastasis.

As BM aspirations are less accepted by patients than taking blood samples, the analyses of genetic alterations in blood from tumor patients might become a particularly attractive approach to assess MRD. For the detection of tumor-specific DNA in blood, the PCR-based microsatellite analysis is a commonly used and specific assay. By this method allelic imbalance of tumor suppressor genes, for example loss of heterozygosity (LOH), can be easily and rapidly determined [13]. The occurrence of LOH, leading to loss of the paired gene product, has been implicated in tumor development, progression and metastases [14]. Our findings have shown that LOH at particular chromosomal loci may reflect tumor cell spread in breast cancer patients [15]. Although a number of studies have evaluated the potential of circulating tumor-associated DNA in blood for the molecular diagnosis and prognosis of various types of cancer [9], the prognostic value of cell-free DNA to identify breast cancer patients at high risk for relapse is largely unknown.

Therefore, the purpose of this study was to study the prognostic relevance of LOH on cell-free DNA at six breast cancer-relevant chromosomal loci in the blood of patients with newly diagnosed breast cancer and to evaluate whether this DNA is a marker of MRD using the presence of DTC in BM as a well-established MRD indicator.

## Materials and methods

### Characterization of study patients and healthy volunteers

The present study was conducted at the Department of Obstetrics and Gynecology at the University Hospital in Essen. In total, 81 patients with primary breast cancer were studied from April 1998 until January 2003. Additionally, 10 healthy female controls aged between 30 and 50 years and with no history of cancer were recruited. Overall survival data of these patients were obtained from the local municipal registry; the median follow-up time was 6.2 years (range 0.2 to 9.8 years). Informed written consent was obtained from all patients, and the study was approved by the Local Essen Research Ethics Committee (05/2856). The clinical data of the patients are summarized in Table 1.

**Table 1:** Patient characteristics at the time of primary diagnosis of breast cancer

	Patients (%)	**LOH (%)**^§^	**DTC (%)**^$^
Total	81	27 (33.5)	32 (39.5)
Age	56 years (range 33-81)		24 (41)
Family history			4 (31)
negative	58 (73)	21 (36)	4 (50)
positive	13 (17)	5 (39)	
unknown	8 (10)	1 (13)	28 (37)
Patient subgroup			
M0	76 (94)	27 (36)	
M1	5 (6)	0 (0)	4 (80)
Tumor size			
pT1	33 (41)	14 (42)	11 (33)
pT2	39 (48)	12 (31)	17 (44)
pT3-4	9 (11)	1 (11)	4 (44)
Nodal status			
pN0	49 (62)	19 (39)	21 (43)
pN_1-2_	30 (38)	8 (27)	10 (33)
Histology			
Ductal	60 (75)	23 (38)	20 (33)
Lobular	10 (12.5)	3 (30)	6 (60)
Others*	10 (12.5)	1 (10)	6 (60)
Grading			
I-II	51 (65)	23 (45)	21 (41)
III	28 (35)	4 (14)	10 (36)
ER status			
negative	23 (28.5)	6 (26)	9 (39)
positive	58 (71.5)	21 (36)	23 (40)
PR status			
negative	29 (36)	8 (28)	8 (28)
positive	52 (64)	19 (37)	24 (46)
CEA			
negative	69 (86)	25 (36)	27 (39)
positive	11 (14)	2 (18)	5 (46)
CA15-3			
negative	67 (84)	25 (37)	27 (40)
positive	13 (16)	2 (15)	5 (39)
Therapy			
BCT	45 (56)	20 (44)	14 (31)
Ablation	36 (44)	7 (22)	18 (50)

*n = 5 tumors were characterized as mixed ductal/lobular carcinomas of the breast; in five tumors histology was unknown. Among these five tumors were three cases of inflammatory breast cancer and one case of a carcinoma sarcoma of the breast with unknown histological subtype.

^§^total loss of heterozygosity (LOH) detected at all six markers in blood

^$ ^disseminated tumor cells (DTC) detected in bone marrow

BCT = breast conserving therapy; CEA = carcino embryonal antigen; ER = estrogen receptor; PR = progesterone receptor.

The median age of the patients was 56 years (range 33 to 81 years). All four initial tumor stages were included, with a predominance of stage I and II. Most patients had ductal breast cancer and 49 women were node negative. High and moderately differentiated tumors were predominant. Two-thirds of the tumors were estrogen receptor (ER) negative and progesterone receptor (PR) positive, respectively. All patients undergoing breast-conserving therapy received an adjuvant radiation. Patients with hormone receptor-positive tumors received an adjuvant hormonal treatment with tamoxifen or an aromatase inhibitor. The chemotherapeutic adjuvant treatment mostly contained anthracyclines and taxanes.

### Immunohistochemical analysis

For each of the 81 patients, the tumor type, TNM-staging and grading were assessed according to the World Health Organization-classification of tumors of the breast [16] and the sixth edition of the TNM Classification System [17]. The ER and PR receptor status were determined by immunohistochemistry.

### Determination of serum tumor markers

For the quantitative determination of carcino embryonal antigen (CEA)/CA15-3 in human serum and plasma, 10 ml serum were collected and assessed using the Elecsys CEA/CA15-3 immunoassays (Roche, Mannheim, Germany). The serial measurement of CEA/CA15-3 was intended to aid in the management of cancer patients. These assays were performed on a Cobas^® ^immunoassay analyzer in the central laboratory of University Hospital in Essen according to the manufacturer's instruction. The central laboratory has a valid certification for the performance of these assays following international guidelines.

### Preparation of bone marrow

BM cells were isolated from heparinized BM (5000 U/ml BM) by Ficoll-Hypaque density gradient centrifugation (density 1.077 g/mol; Pharmacia, Freiburg, Germany) at 400 g for 30 minutes. Interface cells were washed (400 g for 15 minutes) and resuspended in PBS. For the detection of cytokeratin-positive (CK+) cells, 3 × 10^6 ^cells (1 × 10^6 ^per slide and area of 240 mm^2^) from each aspiration side were directly spun (400 g for 5 minutes) onto glass slides coated with poly-L-lysine (Sigma, Deisenhofen, Germany) using a Hettich cytocentrifuge (Tuttlingen, Germany).

### Immunocytochemistry

After overnight air drying, staining of CK+ cells was performed using the Epimet^® ^kit (Micromet, Munich, Germany). The identification of epithelial cells using this kit is based on the reactivity of the murine monoclonal antibody (Mab) A45-B/B3, directed against a common epitope of CK polypeptides. The kit uses Fab fragments of the pan-Mab conjugated with alkaline phosphatase molecules. The method includes: permeabilization of the cells by a detergent (5 minutes); fixation by a formaldehyde-based solution (10 minutes); binding of the conjugate Mab A45-B/B3-alkaline phosphatase to cytoskeletal CKs (45 minutes); and formation of an insoluble red reaction product at the binding site of the specific conjugate (15 minutes). Subsequently, the cells were counterstained with Mayer's hematoxylin for one minute and finally mounted with Kaiser's glyzerine/gelatine (Merck, Darmstadt, Germany) in Tris-EDTA buffer (Sigma, Deisenhofen, Germany). A conjugate of Fab-fragment served as a negative control. For each test a positive control slide with the breast carcinoma cell line MCF-7 (ATTC, Rockville, MD, USA) was treated under the same conditions. The microscopic evaluation was carried out independently by two investigators. Patients were evaluated as tumor cell-positive if at least one CK-positive cell was detected as analyzed by immunocytochemistry.

### Preparation of serum and leukocytes

From each patient, 10 ml whole blood was collected in routine S-Monovette^® ^tubes (Sarstedt AG&Co, Nümbrecht, Germany) and immediately stored at 4°C. Serum and leukocyte preparations were performed within four hours. The blood samples were centrifuged at 2500 g for 10 minutes. The upper phase contained the blood serum, from which 3 to 4 ml was removed for the extraction and analysis of the circulating DNA. The remaining 16 to 17 ml blood was supplemented up to 50 ml with lysis buffer containing 0.3 M sucrose, 10 mM Tris-HCl pH 7.5, 5 mM MgCl_2 _and 1% Triton X100 (Sigma, Taufkirchen, Germany). Following incubation for 15 minutes on ice, the isolation and purification of the leukocytes were carried out by two centrifugation steps at 2500 g, 4°C for 20 minutes.

### Preparation of paraffin-embedded tumor tissue

Tumor tissue of 22 patients was available. Specimens were retrieved from the Institute of Pathology and Neuropathology of the University Hospital of Essen. Tumor pieces of 3 mm in size were processed from paraffin-embedded tumor blocks and embedded in paraffin again to perform six sections of 10 to 20 μm thickness. The sections were dewaxed in 1 ml xylene on a shaker incubator at 45°C for five minutes. After centrifugation at full speed and room temperature for five minutes, the supernatant was removed. Pellets were washed in 1 ml of ethanol and centrifuged at full speed for five minutes. The supernatant was removed, and the pellet was dried at 45°C for two to five minutes until the ethanol had evaporated.

### DNA extraction and fluorescence-labeled PCR

For the PCR-based fluorescence microsatellite analyses we used our former microsatellite method without extended fractionation step [15], because the fractionation technique which separates blood DNA in short and long DNA fragments was not yet established [18] when blood samples were collected between 1998 and 2003.

Genomic DNA was extracted from tumor tissues, leukocytes and serum of peripheral blood using the QIAamp Blood DNA Mini Kit (Qiagen, Hilden, Germany) according to the manufacturer's instructions. Quantification and quality of the extracted DNA were determined spectrophotometrically using the BioPhotometer (Eppendorf, Hamburg, Germany) or the NanoDrop Spectrometer ND-1000 (Peqlab Biotechnologie, Erlangen, Germany). To determine the lowest portion of tumor-specific DNA which can be flawlessly detected, dilution experiments were performed. For this study we mixed and amplified known quantities and proportions of normal leukocyte and serum DNA, as described before [18].

Serum, tumor and leukocyte (reference) DNA were amplified with a PCR using primer pairs binding to microsatellite markers as summarized in Table 2. PCR conditions were described before [15]. To confirm the microsatellite alterations, each PCR was repeated at least twice.

**Table 2:** Microsatellite markers used for loss of heterozygosity analysis

Microsatellite markers	Chromosomal region	Tumor suppressor gene	Function
D3S1255	3p24.2-25	unknown	
D9S171	9p21	P16 (INK4A)	Regulator of cell cycle
D10S1765	10q23.3	PTEN phosphatase and tensin homologue	Regulator of cell growth, metabolism and survival
D13S218	13q12-13	BRCA2 Breast cancer type 2	Regulator of cell cycle
D16S421	16q22-23	E-cadherin	Epithelial cell adhesion molecule
D17S855	17q21	BRCA1 Breast cancer type 2	Regulator of cell cycle

### Evaluation of PCR products

The fluorescence-labeled PCR products were separated by capillary gel electrophoresis and detected on an automated Genetic Analyzer 310 (Applied Biosystems, Freiburg, Germany). Fragment length and fluorescence intensity were evaluated by the GeneScan software. The 500-ROX size marker (Applied Biosystems, Freiburg, Germany) served as an internal standard. The LOH incidence was determined by calculating the ratio of the intensities of the two alleles from a serum or tumor sample corrected by the ratio of the intensities of the two alleles from the corresponding leukocyte sample which served as reference DNA. LOH was interpreted if the final quotient was less than 0.6 or more than 1.67. Homozygous and non-analyzable peaks were designated as non-informative cases.

### Statistical analysis

The statistical analyses were performed using the SPSS software package, version 13.0 (SPSS Inc. Chicago, IL, USA). The chi-square or two-tailed Fischer's exact test, and the univariate binary logistical regression were used to identify possible associations between the occurrence of DTC in BM, DNA concentrations and LOH patterns in blood and the following established risk factors of the patients with primary breast cancer: age, histology results, tumor stage (TNM), nuclear grade, ER and PR status, presence of tumor markers CEA and CA15-3, relapse time, menopause status, family history, and use of chemotherapy, radiotherapy and hormone therapy. In addition, the Mann and Whitney-U and the Wilcoxon-W test for the non-parametric comparison of two independent and dependent variables were used, respectively. Kaplan-Meier plots were drawn on to estimate overall survival and recurrence, and the log rank test was used for statistical analyses. A *P *value of less than 0.05 was considered as statistically significant.

## Results

### Clinical relevance of cell-free DNA concentrations

**Figure 1:**
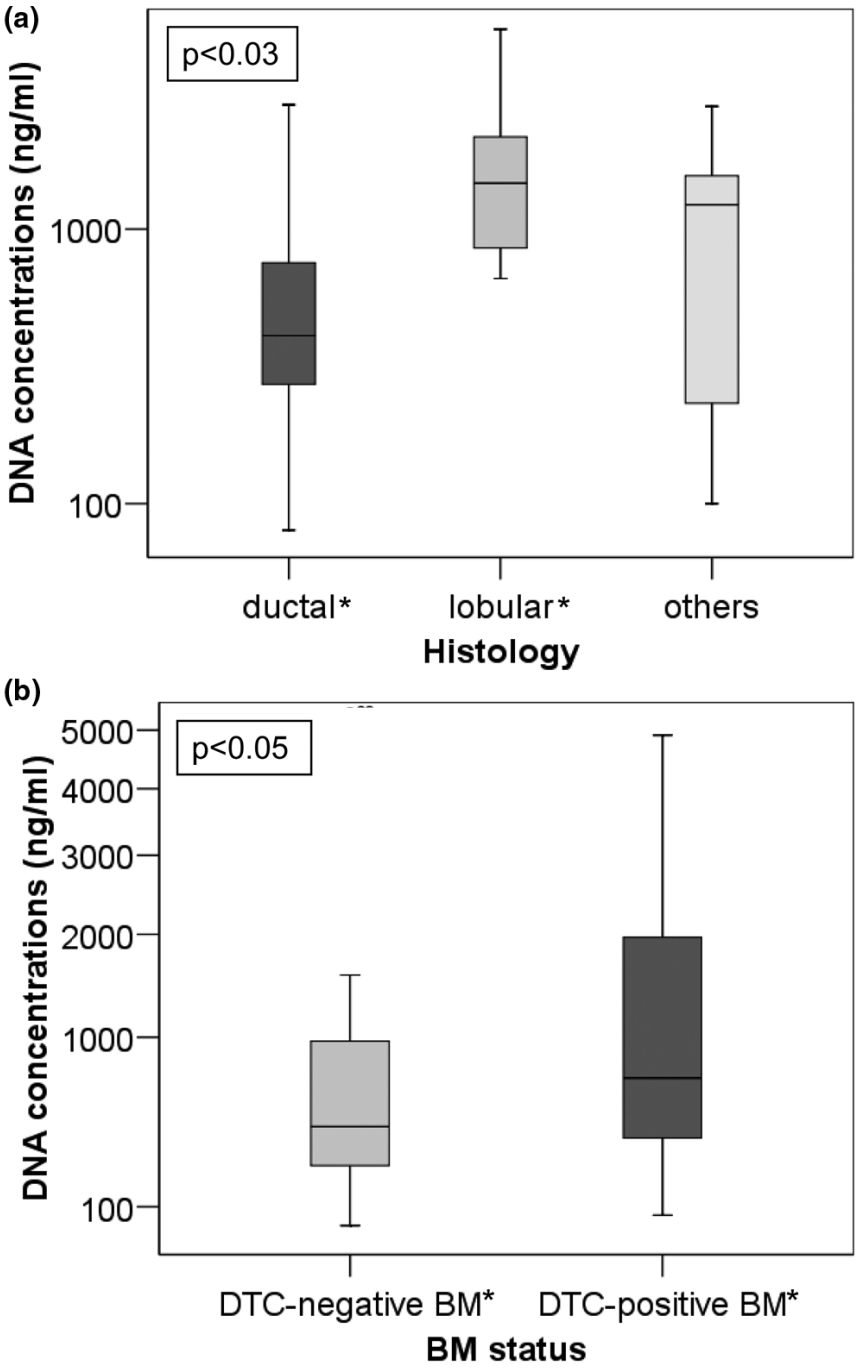
High DNA levels significantly associate with lobular breast cancer. The box plot shows the comparison of serum DNA concentrations **(a) **in patients with ductal (n = 60), lobular (n = 10) and other (n = 10) breast tumors, and **(b) **in patients with disseminated tumor cells (DTC)-negative and DTC-positive bone marrow (BM) status. *Statistical significance according to the Mann and Whitney-U test for the non-parametric comparison of two independent variables.

DNA was extracted from blood serum of 81 breast cancer patients. The range of DNA concentrations was between 58 and 5317 ng/ml of serum with a mean value of 886 ng/ml and a median value of 519 ng/ml. As shown in the box plot of Figure 1a, the patients with lobular breast cancer had significantly higher DNA levels in their blood than patients with ductal breast cancer (*P *< 0.03, Table 3). Patients with DTC-positive BM had higher DNA yields than patients with DTC-negative BM (*P *< 0.05, Figure 1b).

**Table 3:** Significant associations between the presence of DTC, serum DNA yields, LOH frequencies at the microsatellite markers and established risk factors determined by the Mann and Whitney-U test

	Elevated serum DNA	^§^**LOH** complete	^§^**LOH at D3S1255**	^§^**LOH at** D9S171 pos	^$^**DTC**
Age					
< 55 years					
> 55 years	no	no	no	***P *= 0.05**	no
Tumor size					
pT1					
pT2-4	no	no	no	***P* < 0.05**	no
Nodal status					
pN0					
pN1-2	no	no	no	no	no
Metastasis					
M0					
M1	no	no	no	no	***P* < 0.05**
Histology					
Ductal					
Lobular	***P* < 0.03**	***P *= 0.05**	no	no	no
others					
Grading					
I-II					
III	no	***P *= 0.006**	no	no	no
^$^DTC					
Positive	***P* < 0.05**	no	no	no	
Negative					
Recurrence	no	no	***P* < 0.05** *P *= 0.009*	***P* < 0.02** ***P*** **= 0.001**	no

*Log rank test

^§ ^loss of heterozygosity (LOH) detected in blood

^$ ^disseminated tumor cells (DTC) detected in bone marrow

Furthermore, the comparison of the DNA levels in the blood of the tumor patients with those of 10 healthy controls showed that minor DNA yields circulate in blood of healthy individuals. This background level of normal DNA probably results from apoptotic lymphocytes present in the blood sample [13,15]. The range of DNA concentrations of the control group was between 36 and 156 ng/ml of serum with a mean value of 60 ng/ml and a median value of 45 ng/ml (data not shown).

### LOH frequency in tumor samples

A PCR-based fluorescence microsatellite analysis was performed using a panel of six different polymorphic microsatellite markers (Table 2). Tumor tissue specimens of 22 patients were available and served as positive controls. The matched leukocyte DNA (normal DNA) of each patient served as a reference sample. LOH was found in every second tumor tissue (50%). Of the 11 patients, 4 patients showed 1 LOH in their tumor sample, 4 patients showed 2, 1 patient showed 3 and 2 patients showed 4 LOH in their tumor sample (data not shown). The overall LOH incidence in the specimens of all analyzed microsatellite markers was 27.5%.

### LOH frequency in serum samples

**Figure 2:**
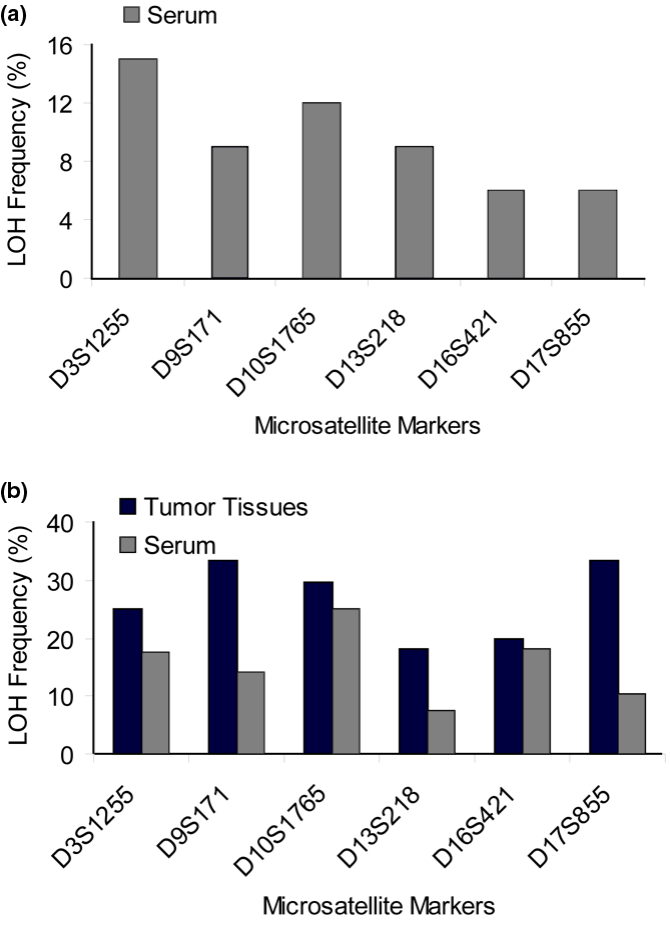
Distribution of the LOH incidences in blood and primary tumor. Comparison of the loss of heterozygosity (LOH) frequencies detected at six different microsatellite markers **(a) **in blood serum samples from 81 breast cancer patients, and **(b) **in serum and tumor samples from 22 patients. The frequency of LOH was calculated by division of the number of LOH with the informative cases for each locus.

Following PCR-based microsatellite analyses on blood serum samples from 81 patients with a panel of six different polymorphic microsatellite markers, at least one LOH was found on cell-free DNA in serum of 27 (33.5%) patients (Table 1). Three patients displayed LOH at two markers in their blood (data not shown). The overall LOH incidence of all analyzed microsatellite markers in blood was 9.5%. Figure 2a depicts the LOH distribution at the different microsatellite markers in the serum samples of the 81 patients. Moreover, blood of the healthy controls did not display LOH at any microsatellite marker used (data not shown).

From 22 patients, both serum and tumor tissues were available. Comparative analyses showed a lower LOH frequency in serum than in tumor tissues (Figure 2b). The LOH distribution of the corresponding serum and tumor samples was heterogeneous and only in three cases was LOH simultaneously detected in both clinical materials, which were all derived from patients with lobular breast cancer (data not shown).

### Detection of DTC in bone marrow

DTC were detected in BM of 32 of 81 patients (39.5%). Four of the five patients with metastatic disease at primary diagnosis were postmenopausal, hormone receptor positive and DTC positive (Table 1). Recurrent disease was diagnosed in 15 of 81 patients (18.5%) after a median of 21 months (range 4 to 54 months), and 22 of 81 patients (27.0%) died after a median of 4.3 years (range 2 months to 9 years). The occurrence of DTC in BM significantly correlated with distant metastases (*P *< 0.05) but with no other clinicopathological factors (Table 3). Both ER-positive and PR-positive patients more often tended to have DTC in their BM than receptor-negative patients (Table 1).

### Clinical relevance of LOH frequency in serum

Statistical evaluations of the LOH frequency in blood from breast cancer patients were performed with the following clinical data: age, family history, tumor size, nodal status, histology, grading, ER and PR status, CEA and CA15-3 (Table 1). Table 3 summarizes the significant associations between these parameters.

The occurrence of LOH at the entire marker set correlated positively with the histologic type of carcinoma (*P *= 0.05) and negatively with the histopathologic grading (*P *= 0.006, Tables 1 and 3). Most LOH were detected in blood of patients with ductal breast cancer (38%) and a histopathologic grading of I to II (45%, Table 1). Statistical evaluations of the LOH frequency at the single markers showed that two markers (D3S1255 and D9S171) were associated with clinicopathological parameters of the patients. The marker D9S171 was only affected by LOH in patients with higher tumor stages of pT2-4 (*P *< 0.05) and patients older than 55 years (*P *= 0.05, Table 3).

**Figure 3:**
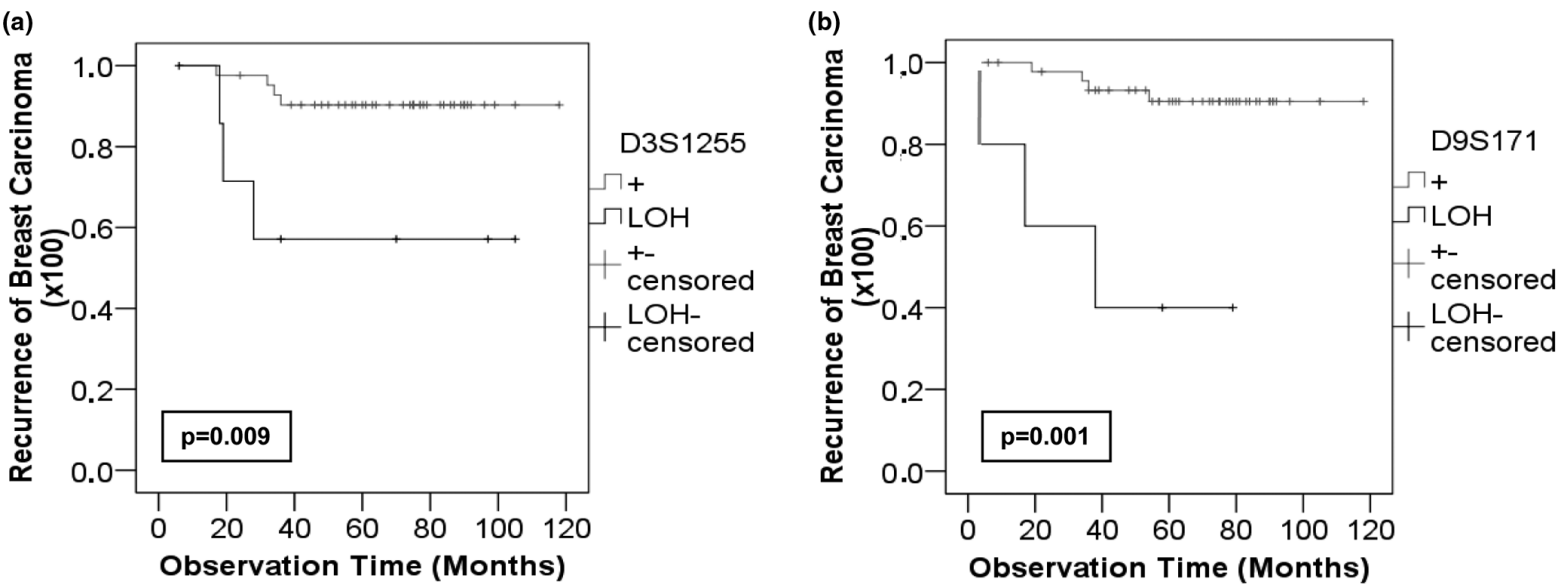
LOH at the marker D3S1255 and D9S171 significantly correlate with the prevalence of recurrence. Kaplan-Meier analyses of recurrent patients with primary breast cancer (n = 76) with the occurrence of loss of heterozygosity (LOH) at the microsatellite markers **(a) **D3S1255 and **(b) **D9S171 at the time of primary diagnosis. Top curves, patients with heterozygosity at the marker D3S1255 (n = 42) and D9S171 (n = 47). Bottom curve, patients with LOH at the marker D3S1255 (n = 8) and D9S171 (n = 5).

In respect to the 76 patients with primary breast cancer without overt metastases, LOH at both markers (D3S1255, *P *< 0.05 and D9S171, *P *< 0.02) significantly correlated with the prevalence of recurrence (Table 3). As estimated by Kaplan-Meier analysis, the median recurrence periods were 109 and 110 months (95% confidence interval (CI) 101 to 117 and 103 to 118) in patients who had no LOH at D3S1255 and D9S171, respectively, whereas the periods were 69 and 43 months (95% CI 39 to 100 and 16 to 71) in patients who displayed LOH at D3S1255 (*P *= 0.009, Figure 3a) and D9S171 (*P *= 0.001, Figure 3b), respectively.

None of the LOH data correlated with a reduced overall survival rate. Whereas nodal status significantly correlated with recurrence (*P *< 0.05) and overall survival (*P *< 0.001), histological grading was associated with a significant shorter overall survival (*P *< 0.03; data not shown).

Finally, we compared the occurrence of LOH at the particular microsatellite markers in blood with the presence of DTC in BM. However, our statistical assessments between LOH and the BM status did not reach any statistical significance for the marker analyzed (Table 3).

## Discussion

In the current study we demonstrated a significant association between cell-free tumor DNA in blood and relapse of breast cancer patients after a follow-up time of 6 to 10 years. Furthermore, the detection of cell-free tumor DNA was not correlated to the detection of DTC in BM as an established indicator of MRD.

We detected markedly higher DNA levels in the blood of patients with lobular breast carcinomas than in patients with ductal tumors, whereas, inversely, the LOH incidence in blood of patients with ductal breast cancer was higher than in patients with lobular cancer. These observations are not easy to explain by an increased turn over of tumor cells because controversial data on the apoptotic and LOH index of the different histological cancer types have been published [19-21]. Furthermore, according to the inadequate number of patients with lobular cancer these data could also not be performed in a multivariate analysis.

Concordant LOH profiles in autologous tumor tissues and blood were only found in 3 of 22 patients where both types of samples were available, whereas discordant LOH profiles were seen in all other cases. This surprising finding may be due to the known multifocal heterogeneity of breast carcinomas [22], the local necrosis of the areas of the primary tumor, the potential contribution of tumor DNA derived from micrometastatic cells to the pool of blood DNA [12] and the masking of LOH caused by the dilution of tumor-derived DNA by normal DNA [15,23].

Nevertheless, we discovered significant correlations between the presence of LOH in blood and the clinical parameters of our patients. The key finding was the association of LOH at the microsatellite markers D3S1255 and D9S171 with the relapse of the patients. In the literature there is little information on the marker D3S1255 mapping to the chromosomal locus 3p24.2-25. However, it has been suggested for another tumor entity that replication errors on 3p is an early event in tumor development and that a tumor suppressor gene, which is involved in the progression of esophageal squamous cell carcinoma, may exist near the 3p25 locus [24]. Moreover, the well-known gene product *CDKN2 *(cyclin-dependent kinase inhibitor) is located in the vicinity of the marker D9S171. *CDKN2 *is a negative regulator of the cell cycle, and the loss of this gene may, consequently, promote cell proliferation, which may also explain its role in the pathogenesis of sporadic breast cancer [25]. Besides the association of LOH at this locus with the relapse of the breast cancer patients, we found that the marker D9S171 was only affected by LOH in patients with higher tumor stages of pT2-4, indicating additionally the relationship between tumor load and the presence of such DNA in serum.

Using a well-established antibody for the detection of DTC [26], we found DTC in BM of nearly 40% of the patients, which is comparable with other published studies demonstrating the prognostic impact of DTC in the BM of primary breast cancer patients [1,27-29]. In our study, the presence of DTC in BM only correlated with distant metastases but with no other clinicopathologic data. Moreover, we found no relationship between BM status and LOH on cell-free DNA in blood. This finding suggests that the presence of DTC in BM of breast cancer patients may not significantly contribute to the release of DNA into blood.

In contrast to DTC, the half life of circulating tumor cells (CTC) seems to be short (1 to 24 hours) and apoptotic cells significantly contribute to the circulating tumor cell fraction in breast cancer patients [30]. Genomic analyses at the single-cell level have shown that DTC and CTC frequently display very heterogeneous tumor-specific aberrations, particular in patients with early-stage cancer without overt metastases [31,32]. Furthermore, genomic and phenotypic differences between DTC, CTC and the primary tumor have been documented in breast cancer [33,34]. As a result, it has been suggested that tumor cells disseminate in an early genomic state and that they acquire genomic alterations after dissemination independent form the primary tumor [35]. Thus, it can be speculated that part of the DNA found in blood is derived from CTC which could also explain the discrepancies in LOH patterns observed between blood and autologous tumor tissues. We did not perform CTC analysis in the current study because no standardized method for CTC screening had been established when samples were taken 6 to 10 years ago. Future genomic analyses of CTC and DTC together with the assessment of cell-free DNA will shed more light on the origin of cell-free DNA in cancer patients.

## Conclusions

The current study assessed the clinical relevance of LOH at particular chromosomal regions on cell-free DNA circulating in the peripheral blood of breast cancer patients, in particular in the context of BM micrometastasis. The detection of LOH on cell-free blood DNA was not related to the presence of DTC in BM but showed a significant independent association to breast cancer recurrence. The molecular analysis of cell-free tumor DNA in blood may contribute to the identification of breast cancer patients with an increased risk for relapse.

## Abbreviations

BM: bone marrow; CEA: carcino embryonal antigen; CI: confidence interval; CK+: cytokeratin-positive; CTC: circulating tumor cells; DTC: disseminated tumor cells; ER: estrogen receptor; LOH: loss of heterozygosity; Mab: monoclonal antibody; MRD: minimal residual disease; PBS: phosphate-buffered saline; PCR: polymerase chain reaction; PR: progesterone receptor.

## Competing interests

The authors declare that they have no competing interests.

## Authors' contributions

HS, BK and SK performed all experiments. HS performed the statistical analysis. HS and SK drafted the manuscript and KP revised the manuscript. FO and RK prepared the clinical material. SK summarized the clinical parameters. HS, SK and KP were involved in conception and design of the study and participated in the discussion and interpretation of results.
